# Association of genetic variations and gene expression in a family-based study

**DOI:** 10.1186/s12919-016-0014-0

**Published:** 2016-10-18

**Authors:** Achilleas N. Pitsillides, Seung-Hoan Choi, John D. Hogan, Jaeyoung Hong, Honghuang Lin

**Affiliations:** 1 National Heart Lung and Blood Institute’s Framingham Heart Study, 73 Mt. Wayte Avenue, Suite 2, Framingham, MA 01702 USA; 2 Department of Biostatistics, Boston University School of Public Health, 677 Huntington Avenue, Boston, MA 02115 USA; 3 Program in Bioinformatics, Boston University, Boston, MA 02215 USA; 4 Department of Medicine, Boston University School of Medicine, 72 East Concord St, Boston, MA 02118 USA

## Abstract

**Background:**

Expression quantitative trait locus (eQTL) maps are considered a valuable resource in studying complex diseases. The availability of gene expression data from the Genetic Analysis Workshop 19 (GAW19) provides a great opportunity to investigate the association of gene expression with genetic variants in blood.

**Methods:**

A total of 267 samples with gene expression and whole genome sequencing data were employed in this study. We used linear mixed models with genetic random effects along with a permutation procedure to create an eQTL map. The eQTL map was further tested in terms of functional implication, including the enrichment in disease-related variants and in regulatory regions.

**Results:**

We identified 22,869 significant eQTLs from the GAW19 data set. These eQTLs were highly enriched with genetic loci associated with blood pressure and DNase hypersensitive regions. In addition, the majority of genes associated with eQTLs showed moderate to high heritability (*h* ^2^ > 0.4).

**Conclusions:**

We successfully created an eQTL map from the GAW19 data set. Our study indicated that the eQTLs were enriched within regulatory regions, and tended to have relatively high heritability.

## Background

Gene expression is an essential component of the central dogma of molecular biology, and is mediated by both genetic and environmental factors [1]. Expression quantitative trait loci (eQTLs) are genomic loci that regulate gene expression. Previous studies have found that eQTLs are enriched with disease-related variants uncovered by genome-wide association studies (GWAS) [2], suggesting that eQTLs might be a good resource to better understand the functional implication of GWAS signals, and uncover potential molecular mechanisms underlying disease etiology. The availability of gene expression data in the Genetic Analysis Workshop 19 (GAW19) provided us an opportunity to investigate the association of gene expression with genetic variants identified through whole genome sequencing [3].

## Methods

### Data description

For each analysis we used real phenotype data from individuals in the Type 2 Diabetes Genetic Exploration by Next-generation sequencing in Ethnic Samples (T2D-GENES) whole genome sequencing (WGS) families provided by GAW19, which included systolic and diastolic blood measurements, age, sex, and smoking status. These measurements were quantified in four visits, and data for at least one visit was available for 939 participants. A full description of the data can be found in Almasy, et al. [4].

The genotype data was generated from WGS, which included 12,296,048 single-nucleotide variants (SNVs) on odd-numbered chromosomes found in at least one of 464 studied participants.

The gene expression profiled was previously described in detail by Göring, et al. [5]. Briefly, peripheral blood was collected from 1,240 individuals. However, only 267 samples also had their genotype profiled by WGS and thus were chosen for our current study. The RNA was extracted from mononucleated cells, amplified, and then hybridized to an Illumina Sentrix Human Whole Genome (WG-6) microarray, which measured the expression profiles of more than 20,000 transcripts. The expression data were collected at the first visit, so we only use trait data and covariates from that visit. Because only 34 individuals used blood pressure medication and we did not expect different types of medication to have the same effect on gene expression, we did not adjust for blood pressure medication use.

### Annotation

The functional annotation of genetic variants was performed using the ANNOVAR (Annotate Variation) package [6]. We defined putative *cis*-eQTLs as SNPs located within 1 Mb of the transcript starting sites, and *trans*-eQTLs as SNPs located more than 1 Mb of the transcript starting sites, or in different chromosomes.

### Expression quantitative trait locus analysis

The association of genetic variants with gene expression (eQTL) was tested by linear mixed effects models, adjusting for random genetic effects and age, sex, smoking status and the first two components from the expression principal components analysis (*Pc*1, *Pc*2) as covariates. Using the subscript *ij* to denote the *j* ^*th*^ individual in the *i* ^*th*^ family, and defining *Y*_*ij*_ and *SNP*_*ij*_ as the gene expression and genotype dosage, respectively, we write the model as:

$$ {Y}_{ij}={\beta}_o+{\beta}_1 ag{e}_{ij}+{\beta}_2se{x}_{ij}+{\beta}_3 smokin{g}_{ij}+{\beta}_4Pc{1}_{ij}+{\beta}_5Pc{2}_{ij}+{\beta}_{s\ }SN{P}_{ij}+{\alpha}_{ij}+{\varepsilon}_{ij} $$

where the betas denote the regression coefficients for the fixed effects, *α*_*ij*_ is the random intercept, and *ε*_*ij*_ the normally distributed error term with mean zero and variance *σ*_*ε*_^2^. The *α*_*ij*_ within the *i* ^*th*^ family are normally distributed with mean zero and covariance matrix *σ* ^2^ *Φ*, where *Φ* is the kinship matrix. The overall covariance matrix is block diagonal with one block per family. The model was fitted in R using the *lmekin* function from the *coxme* package [7]. The inclusion of the principal components in the model is to account for batch effects.

Because of small sample size (N = 267), we had limited power to uncover *trans*-eQTLs. In particular, with our pedigree structure, assuming gene expression is fully heritable, we have 30 % power to detect a common SNP that would explain 0.2 of the variance at a significance level of *α* = 1*E* − 8. Consequently, this study concentrated on *cis*-eQTLs, which were defined as SNPs within 1 Mb from the start or end of the transcript [8].

We ran a Shapiro-Wilk normality test for all the expression probes and we found that the test was significant after a Bonferroni correction for more than half of the probes. To reduce type I error that may arise from violating the normality assumption, we estimated significance using a modified version of the permutation procedure proposed by Iturria, et al. [9] that preserves familial correlation. First, we estimated the heritability for each transcript and binned the transcripts according to the estimated heritability. For each transcript, we picked a surrogate from the same heritability bin to represent the expected association that we would have observed with a random transcript unassociated with the SNP of interest. We then reordered the original transcript values according to the ranking of the surrogate gene values. We reran the analysis with the permuted phenotype values and the putative *cis*-eQTL for the original transcript. We repeated the above procedure for each transcript three times to obtain a *p* value between *cis*-eQTL and transcript.

To obtain the significant results we split the genes into seven heritability bins. This splitting choice gave an approximately equal number of permutations per bin. To adjust for the different number of SNPs tested (*N*_*g*_) for each gene and account for linkage disequilibrium, we multiplied the *p* values by the factor $\frac{N_g+1}{2}$, which has been shown to be a good estimate of the number of effective tests [10]. We used the minimum adjusted *p* value from each permutation to form a null distribution of the adjusted *p* value statistic for each heritability bin.

### Enrichment of eQTLs in disease-related variants and regulatory regions

We took all the significant eQTLs and performed a Fisher’s exact test to determine if the eQTLs were significantly enriched with known genetic loci associated with blood pressure. A total of 26 top SNPs were collected from the literature. However, none of these SNPs were eQTLs found in this study, either because they were not directly sequenced, or because they were rare variants (minor allele frequency (MAF) <5 %). Therefore, we extended this list to nearby SNPs with distance less than 1 kb and *r* ^2^greater than 0.9. This extended list contained 31 SNPs that were also eQTLs in our study.

We also examined if eQTLs were significantly enriched within DNase hypersensitive regions based on ENCODE data [11].

## Results

### Expression quantitative trait locus map

Here, we report significant expression SNPs (eSNPs) controlling for a false discovery rate of 0.05 using the Benjamini-Hochberg procedure [12]. We found 21,753 significant eSNPs that controlled the expression of 332 distinct Genes (eGenes); 985 eSNPs targeted more than one eGene. Only one eGene has low heritability (*h* ^2^ < 0.1), and all the other eGenes have medium to high heritability (*h* ^2^ > 0.4). The ten most significant eQTLs are shown in Table 1 and the breakdown of the results by heritability bin is shown in Table 2.

**Table 1:** Top 10 eQTLs

Gene symbol	Chr	Pos	Ref	Alt	Raw *p* value	Adjusted *p* value
*UBA52*	19	18668135	G	C	<1e-324	<1e-324
*UTS2*	1	7970383	T	C	<1e-324	<1e-324
*CAPZA1*	1	113138318	G	A	1.9e-150	3.0e-147
*TMEM176B*	7	150478052	G	T	1.9e-139	3.2e-136
*TMEM176A*	7	150460891	T	G	1.7e-134	3.0e-131
*RPL14*	3	40498845	T	C	3.3e-123	4.4e-120
*TIMM10*	11	57324428	G	A	1.2e-120	1.9e-117
*SPATA20*	17	48624523	A	C	1.8e-112	2.7e-109
*SLC12A7*	5	1104938	C	T	4.0e-97	7.7e-94
*RNF167*	17	4848450	G	A	1.1e-92	2.2e-89
*RPS6KB2*	11	67197757	G	A	3.9e-90	2.9e-87

**Table 2:** Summary results by *h* ^2^ bin

h^2^	eSNPs	Genes tested	SNPs per genes (mean)
0–0.1	4	451	3253
0.1–0.2	0	580	3111
0.2–0.3	0	800	3198
0.3–0.4	0	1145	3141
0.4–0.5	2232	1272	3210
0.5–0.6	350	1079	3123
0.6–1	20283	904	3177

### Enrichment of expression quantitative trait loci in blood pressure–related variants

To demonstrate the usefulness of our eQTL map, we examined the distribution of blood pressure–related genetic variants from GWAS found in the literature. Our variant list contained 31 SNPs that were a part of the eQTL study, two of which were eSNPs. Even with the small numbers, a Fisher’s exact test (*p* <6.1E-4) showed that the set of GWAS results contained more eSNPs than we would expect by chance.

### Enrichment of expression quantitative trait loci in regulatory regions

We found 386,135 variants that were part of the eQTL analysis that lie within DNase hypersensitivity regions, of which 5,679 were found to be eSNPs. Our results confirmed the enrichment of eQTLs in DNase cluster regions (Fisher’s exact test *p* value <2.2e-16), which contained 17 times more eQTLs than random variants.

### Association of gene expression with blood pressure

We then tested the association of gene expression with blood pressure, and found that two probes were significantly associated with blood pressure. One probe (GI_7706275.A with *p* <5.5E-9) maps to the gene *TPP3* on chromosome 16, while the other (GI_42661149 with *p* <1.8E-6) maps to the predicted gene *LOC400604*.

## Discussion

The identification of genetic mechanisms underlying gene expression would enable a better understanding of the biology of complex diseases. We performed a comprehensive analysis of the association between gene expression and genetic variants using GAW19 data, and successfully identified 22,869 eQTLs despite limited sample size. These eQTLs were highly enriched in genetic loci associated with blood pressure and DNase hypersensitive regions, suggesting that eQTLs might play an important role in regulatory functions. Our breakdown of eSNP results by heritability indicates that most of these eSNPs have high heritability. Future investigation with a larger sample size would further validate the association of heritability with eQTLs.

## Conclusions

We created an eQTL map that included 22,869 eQTLs from GAW19 data. We found that eQTLs were enriched within regulatory regions, and tended to have relatively high heritability.
